# Turning data into better mental health: Past, present, and future

**DOI:** 10.3389/fdgth.2022.916810

**Published:** 2022-08-17

**Authors:** Nidal Moukaddam, Akane Sano, Ramiro Salas, Zakia Hammal, Ashutosh Sabharwal

**Affiliations:** ^1^ Department of Psychiatry, Baylor College of Medicine, Houston Texas, United States; ^2^ Department of Electrical and Computer Engineering, Rice University, Houston, Texas, United States; ^3^ Department of Psychiatry, Baylor College of Medicine, The Menninger Clinic, Michael E DeBakey VA Medical Center, Houston, Texas, United States; ^4^ The Robotics Institute Department in the School of Computer Science, Carnegie Mellon University, Pittsburgh, PA, United States

**Keywords:** bio-behavioral sensing, apps and smartphones, ecological momentary assessment, mental health, craving and relapse, anhedonia, depression

## Abstract

In this mini-review, we discuss the fundamentals of using technology in mental health diagnosis and tracking. We highlight those principles using two clinical concepts: (1) cravings and relapse in the context of addictive disorders and (2) anhedonia in the context of depression. This manuscript is useful for both clinicians wanting to understand the scope of technology use in psychiatry and for computer scientists and engineers wishing to assess psychiatric frameworks useful for diagnosis and treatment. The increase in smartphone ownership and internet connectivity, as well as the accelerated development of wearable devices, have made the observation and analysis of human behavior patterns possible. This has, in turn, paved the way to understand mental health conditions better. These technologies have immense potential in facilitating the diagnosis and tracking of mental health conditions; they also allow the implementation of existing behavioral treatments in new contexts (e.g., remotely, online, and in rural/underserved areas), and the possibility to develop new treatments based on new understanding of behavior patterns. The path to understand how to best use technology in mental health includes the need to match interdisciplinary frameworks from engineering/computer sciences and psychiatry. Thus, we start our review by introducing bio-behavioral sensing, the types of information available, and what behavioral patterns they may reflect and be related to in psychiatric diagnostic frameworks. This information is linked to the use of functional imaging, highlighting how imaging modalities can be considered “ground truth” for mental health/psychiatric dimensions, given the heterogeneity of clinical presentations, and the difficulty of determining what symptom corresponds to what disease. We then discuss how mental health/psychiatric dimensions overlap, yet differ from, psychiatric diagnoses. Using two clinical examples, we highlight the potential agreement areas in assessment/management of anhedonia and cravings. These two dimensions were chosen because of their link to two very prevalent diseases worldwide: depression and addiction. Anhedonia is a core symptom of depression, which is one of the leading causes of disability worldwide. Cravings, the urge to use a substance or perform an action (e.g., shopping, internet), is the leading step before relapse. Lastly, through the manuscript, we discuss potential mental health dimensions.

## Introduction

The saying, “knowledge is power”, widely credited to Sir Francis Bacon (Meditationes Sacrae (1597)), is very aptly suitable to today's data-rich landscape. Data collected from phones, apps, and other technology is staggering in extent. If knowledge is power, then data is king. In this review, we will present an interdisciplinary overview of how data can be used for the assessment and betterment of mental health.

Smartphone ownership and internet connectivity have been increasing worldwide. It is estimated that 77% of adults in the US have an internet-enabled smartphone, with rates close to 100% for the age group 18–29 (1). Rates of smartphone ownership and internet connectivity seem to follow general trends: younger individuals have the highest smartphone ownership, and the age gap is closing since 2015, with older adults also acquiring smartphones. The ubiquitous presence of technology allows an unprecedented glimpse into an individual's life from privately spent moments to those spent with other people. Smartphones often play the role of a wearable and can be used to track behavioral patterns throughout the day, as discussed below.

Wearables/apps can measure human behavioral patterns that can be predictive of symptom resurgence, and can, hypothetically, provide diagnostic adjuncts and potential treatments. However, to achieve full potential, data has to be translated into meaningful, actionable input that links to what is currently known about mental health and psychopathology. In this paper, we will discuss how data collected from wearables/smartphones can be leveraged for a better understanding of mental health conditions and used to improve mental health. We will start by outlining what data can be collected (section II), what it could correspond to in current psychiatric terminology (section II), and what to seek as a ground truth reference for collected parameters, such as functional imaging (section III). We will then apply the outlined principles, as examples, to concepts such as cravings and anhedonia (section III). These two concepts were selected because they exemplify the spectrum of feeling to behavior that is challenging to define, yet perfectly suited for the kinds of technology we will be discussing.

We would be remiss if we did not mention socio-economic disparities and mental-health-based disparities, by which any patients with mental illness are in the bottom rungs of society and do not have the means to have a smartphone/wearable. Additionally, individuals in emerging countries may have less access to smartphones and internet connectivity. Thus, this review focuses on those who already have access to this technology, and how to optimize the use of their available data.

It is also of note that the field of behavioral sensing and using app/wearable data is much better developed in specialties related to medical conditions and surgical procedures than to mental health conditions. For instance, in planning discharge from hospital post-surgical procedures, using a smartphone app helps better management by improving pain management, early detection of complications, and reducing unscheduled visits (1). The field of wearable/app use in mental health is still developing because of (1) diagnostic challenges, (2) lack of concordance between patient-generated and clinician-generated assessments, and (3) lack of objective biomarkers for measurement and tracking of mental health conditions. These points are elaborated in the next paragraphs.

Psychiatric diagnoses follow different frameworks; the Diagnostic and Statistical Manual, first edition, was released in 1952 and has gone through multiple iterations to reach the current DSM-5 (2), whereas the International Classification of Disease (ICD) started in 1893. The current ICD-11 (3) has about 55,000 diagnostic codes. Despite attempts at reconciliation, DSM-5 and ICD-11 still diverge on some mental illness definitions. With the advent of big data, collected from wearable technology and smartphones, there is a need to reconcile data interpretation with diagnostic categories as well, otherwise achieving usefulness in diagnosis, follow-up, and treatment for mental illness would not be possible.

Dimensional hierarchical classifications in psychiatry seem to offer a reasonable counterpart to technology-based data collection, but the discussion about dimensional categories to adopt, the pros and cons of each system, is still ongoing. Well stated by Arseneault et al (4), “A productive debate about the appropriateness of a categorical diagnostic system is still ongoing, and concerted scientific efforts have resulted in proposals for sophisticated models as alternative approaches to psychiatric nosology, including the Hierarchical Taxonomy of Psychopathology (HiTOP), the transdiagnostic approach and the Research Domain Criteria (RDoC)”. The widely used DSM-5 has been harshly criticized as the categorical disorders are simply groups of symptoms that tend to go together. For example, in major depressive disorder (MDD), there are 9 categories (depressed mood, anhedonia, suicidal thoughts, poor sleep, either too much or too little, etc.) and if a patient answers yes to 5 of those 9 (and those 5 include anhedonia and/or depressed mood) then the patient is diagnosed with MDD. The advantages are that DSM provides a common language and that it provides a categorical diagnosis so clinicians can prescribe antidepressants and insurances will pay for such treatments. The disadvantages are that MDD is not a real disorder, as two patients may share no symptom, yet they will likely receive the same treatment. The RDoC tries to fill this gap by studying psychiatry dimensionally and by symptoms instead of diagnoses. In addition, the RDoC created a “matrix” in which constructs (such as positive valence, negative valence, cognition) and units of analysis (cells, genes, behavior, etc.) are suggested as possibly important to be studied. However, the RDoC is a framework for research, not yet the start of new psychiatry. Interdisciplinary research is needed in this area, to bridge psychiatry and computer sciences/engineering.

Therefore, to properly utilize the vast amounts of data available to researchers, a first step, as covered in the next section, is to understand (1) what information sensors provide, (2) how this meshes with patients' self-reports, and (3) how this data links to observable, detectable actions. Psychiatry has long dealt with the discrepancy between self-report and objective assessments. The answer has been combining self-report measures (e.g. PHQ-9 for depression) and clinician-assessed measures (e.g. Hamilton Depression scale), but as a whole, the discipline lacks objective, widely used disease biomarkers. Hierarchal, dimensional models are suited for studying psychiatric symptoms with a combination of social aspect, neurobiological aspect (imaging+ genetic/epigenetic markers), and approximate clinical descriptors. Technology-based data would then be the added piece in this puzzle, and can bridge the gap between feelings (collected by ecological momentary assessment, EMA), and actions, detected by sensors or reported by the patient.

## Usefulness of technology- what do we measure and how?

Recent advances in Internet-connected wearable and mobile technologies allow individuals to monitor their daily lives while researchers passively collect long-term real-time physiological and behavioral data without disrupting human routines. Data from personal digital devices may be used to understand users' mental health and behaviors and suggest actions and contents to enhance mental health states. For example, current emotional status, behavior history, and potential future emotion trajectories information might help (1) individuals become more aware of their risk profiles and enable them to make better informed decisions and take actions to change their behaviors to reduce potential negative physical and mental outcomes, and (2) computers to suggest actions or contents that might help users to enhance users' mental health.

### Biobehavioral data for mental health

**Figure 1:**
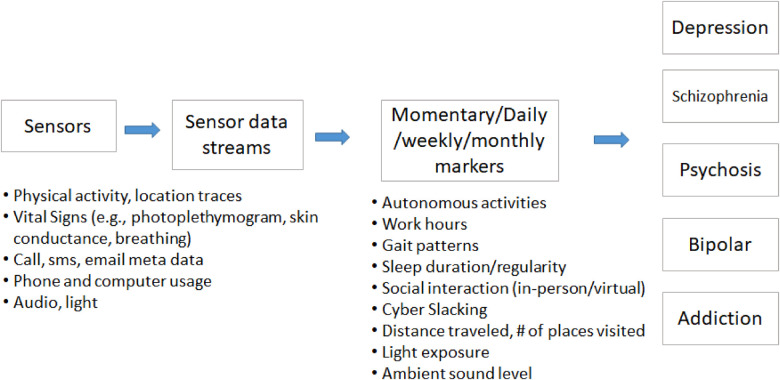
Example of a layered, hierarchical sensor-based framework.

Different kinds of bio-behavioral data have been used to study the relationships with mental health symptoms (Figure 1). These objective measures have potential to help screen, diagnose, and track mental health disorders. The data include (1) non-verbal behavior such as facial activities, body gestures, eye moments, posture, pupil dilatation, (2) speech features, (3) text, and (4) mobile or wearable sensor-based physiological and behavioral signals.

### Non-verbal visual information

Automatic depression assessment has been studied using facial expression, action units, eye movement, and head post and orientation (5–13). There are some public audio-visual databases with self-report or clinical depression assessments (e.g., Diagnostic and Statistical Manual of Mental Disorders fourth edition (DSM-IV), The Hamilton Depression Rating Scale (HAM-D)). These data were collected during (1) interactions with a clinical psychologist or family members, or (2) experimental sessions for eliciting emotions using stimuli such as video clips or images selected from the International Affective Picture System (IAPS). Machine learning models have been developed for detection, severity assessment, and prediction of depression. Multiple contributions to multimodal computational approaches for automatic measurement of depression severity in clinically relevant participants have been proposed. Most previous efforts in automatic assessment of depression severity have focused on facial expression (7, 8), voice quality and timing (9), head pose (7, 8, 10), and body movement (11, 12). For instance, Hdibeklioglu and colleagues (7) proposed a state of the art multimodal (face, head, and voice) deep learning based approach to detect depression severity in participants undergoing treatment for depression. The dynamics of facial, head, and vocal prosody was important for the measurement of depression severity, but one could not say whether the dynamics of those measures was increasing, decreasing, or varying in some non-linear way. To overcome that limitation, Kacem and colleagues (8) proposed a method to measure depression severity from facial and head movement dynamics using affine-invariant barycentric and Lie algebra representation, respectively. Consistent with clinical data, the extracted kinematic features revealed that the velocity and acceleration of facial movement strongly mapped onto depression severity. Body movement also changes with depression severity (e.g., inability to sit still, slowed body movement [DSM-5]). In Joshi and colleagues (11), the authors investigated body movement for the detection of depression using the relative orientation and radius of body parts as well as the holistic body motion measured using space-time interest points. The two sets of descriptors were then fused to train a support vector machine (SVM) classifier for the detection of presence from the absence of depression. From a clinical perspective, it is also critical to measure change over time in depression severity. In the continuity with these efforts, Daoudi and colleagues (12) investigated the discriminating power of body movement dynamics for depression severity assessment. To capture changes in the dynamics of body movement that would reflect the psychomotor retardation and agitation of depressed participants, Gram matrices formulation was used for body shape and trajectories representation. Relevant kinematic features were then extracted from body shape trajectories (i.e., velocities and accelerations). Gaussian Mixture Models (GMM) combined with an improved fisher vector encoding were then used to obtain a single vector representation for each sequence (i.e., clinical interview). Finally, a multi-class SVM with a Gaussian kernel was used to classify the encoded body movement dynamics into three depression severity levels: severe, mild, and remission.

### Speech

Speech features such as prosodic, source, and acoustic features, as well as vocal tract dynamics signals have been studied to assess mental health conditions focused on depression, bipolar disorder, and schizophrenia. Open-access research data sets have been distributed at Audio/Visual Emotion Challenge and Workshop (AVEC) machine learning competitions and promoted the development of machine learning models (13). In addition, recent work has proposed two new measures that are computed using modern deep learning-based analysis of free-living speech. First is social ambiance (14) that captures the level of background speech activity as a proxy for types of environments chosen by an individual. Social ambiance levels are found negatively associated with Neuroticism personality for depression patients, and GAD-7 for psychosis patients (*p* < 0.05). The second measure is called In-person Social Network (IPSN) size (15) which measures how many unique speakers an individual interacts with. IPSN size was significantly correlated with depression severity assessed using PHQ-9 scores (*r* = −0.56, *p* < 0.01).

### Text

Digital text data on emails or social media have also been studied for assessing mental health such as depression, mood valence, and suicide ideation (16). The data are processed to extract linguistics features such as emotionally positive, neutral, and negative words, as well as behavioral features such as the number and timing of tweets and the number of connections. If text messages are analyzed in a privacy-preserving manner, without linguistic analysis, then the length of the messages and the timing is found to correlate with anxiety and depressive symptoms (17, 18).

### Physiology, environment, behavior, and patient data

Mobile or wearable sensors or devices have been used for sensing the following six categories of data:

(1) Physiology: skin conductance, skin temperature, and photoplethysmography (PPG) using a smartwatch (e.g., AppleWatch, Wavelet wristband, Empatica E4) or electrocardiogram from a chest sensor.
(2) Location and Movement: GPS and accelerometer data to measure location and movement.
(3) Communication: App usage (which apps were used for how long), call and text messages (who called/texted when and the length of the messages/calls, but not the content).
(4) Environment: Ambient audio features such as ambient sound volumes, frequency, and weather data (e.g., temperature and humidity) based on the location data.
(5) Ecological Momentary Assessment (EMA): emotion labels (e.g. sad-happy, positive and negative affect (19, 20), depressive mood, and stress) are defined as binary (high/low), three class (high/mid/low), Likert-scale or continuous (e.g., 0–100 scale). Other data such as daily activities (e.g., academic and work schedule, social interaction, sleep diaries). EMA data are sampled a few times per day or twice a day (morning and evening) depending on the studies.
(6) Patient/User profile: this category relies on self-report but has also been studied in conjunction with some of the major self-report, validated questionnaires in the field of psychiatry. Areas covered include gender, personality type according to the big five personality classification (21), perceived stress as measured by the perceived stress scale (PSS) (22), mental health scores according to HAM-D, PHQ-9, GAD-7, the 12-item short form health survey (23), and other clinical assessment.

### From data to biobehavioral markers and mental health

The data collected in daily life settings can be sampled every few milliseconds to every few days. These data can be noisy and include missing segments due to various reasons (e.g. paused sensor data collection, sensor power outage) and they require preprocessing such as filtering noise, interpolating missing data, or extracting features/markers effectively. Some tools have been developed to automatically detect noise on physiological data such as electrocardiogram, photo-plethysmogram, and skin conductance (24). Checking the data quality while collecting the data is crucial to collect higher quality data (25).

Data collected can be a snapshot into a short event in a person's day, or aim to capture more complex interactions. Establishing a physiological or behavioral baseline for each patient or a group of patients becomes the stepping stone/ basis for recognizing signs and symptoms of mental illness *via* deviations from established patterns possible. Researchers developed algorithms or machine learning models to detect behavioral and physiological markers from sensor samples. These markers include autonomous responses (heart rate, time domain or frequency domain heart rate variability index, in person or call, email, or SMS social interaction, app usage, conversation duration, sleep duration and regularity, work hours, hours home, cyberslacking, distance traveled. Previous studies have analyzed the relationships between these markers and the symptoms or severity of mental health disorders or develop machine learning models to detect or predict the severity of mental health symptoms using markers or sensor signals (26).

Here, we introduce two examples of biobehavioral markers and the relationship with mental health in order to progress from sensor-based data to higher-order concepts encompassing several data modalities linked in an attempt to understand human behaviors.

#### Behavioral rhythmicity

When blending results from multiple sensor modalities, it is essential for each person to become their own reference, or baseline, as each individual has different baselines and routines (e.g. meal intake, sleep, exercise, work schedules). Sensor-based data collection is suited for this type of assessment that can later be analyzed by machine learning algorithms. This type of data collection can easily exceed self-report in usefulness as one of the main issues in mental illness remains lack of insight and lack of proper objective assessment of one's won symptomatology.

As an example, we quantified sleep regularity index as a probability of being in the same state (asleep or awake) at any two points 24 h apart and daily routineness based on GPS data and found that these markers are related to mood (25, 27). In addition, we quantified behavioral rhythms from mobile phone sensor data for schizophrenia patients and found that we can predict schizophrenia patients' self-reported symptoms and relapse events using behavioral rhythmicity features (e.g. ultradian rhythm: rhythm shorter than 24 h, circadian: around 24 h, and infradian: longer than 24 h) (28–30). This type of analysis is truly transdiagnostic, as it reflects an individual risk of symptom resurgence regardless of diagnostic category.

#### Sociability

Sociability is the tendency an individual has to affiliate with others. In the field of psychiatry, sociability is rarely examined as an objective quality and is rather looked at from a pathological perspective (31–34) (loneliness, isolation due to depression or negative symptoms of schizophrenia, etc.). Recently, research has turned to examine the social network of an individual, rather than their assessment of it, a crucial step into dimensional hierarchical psychiatric conceptualization. Gianfredi et al (35), for instance, have found a link between annually assessed depressive symptoms and the size of an individual's social network in the Maastricht Study, a population-based prospective cohort study, suggesting an impoverished social network may be linked to more depression.

The author's team has taken the approach of examining the number of individuals a person spends time with and their proclivity to crowded versus quiet places as measures of sociability, with the goal of delving further into the unique contribution of the speaker and how it links back to the development of psychopathology.

We have quantified sociability as a proxy for overall social activity in the environment based on the number of simultaneous speakers by processing unconstrained audio recordings collected from wrist-worn audio-bands (36). We compared sociability patterns and time spent at four different ambiance levels between patients with depressive or psychotic disorders and healthy controls. Our analysis showed that patients with depression/psychosis spent less time in diverse environments and less time in moderate/active ambiance levels; they also spent more time alone. In addition, social ambiance patterns were related to the severity of self-reported depression, anxiety symptoms, and personality traits. Machine learning has already allowed some insight into how two people connecting in a therapy session can be used to help predict therapy outcomes (37). This type of research is an example of how objective measurements can later provide a larger framework for developing future treatments, such as evidence-based social skills training, or may even the far-reaching goal of mitigating the development of negative symptoms in first-episode psychosis.

**Figure 2:**
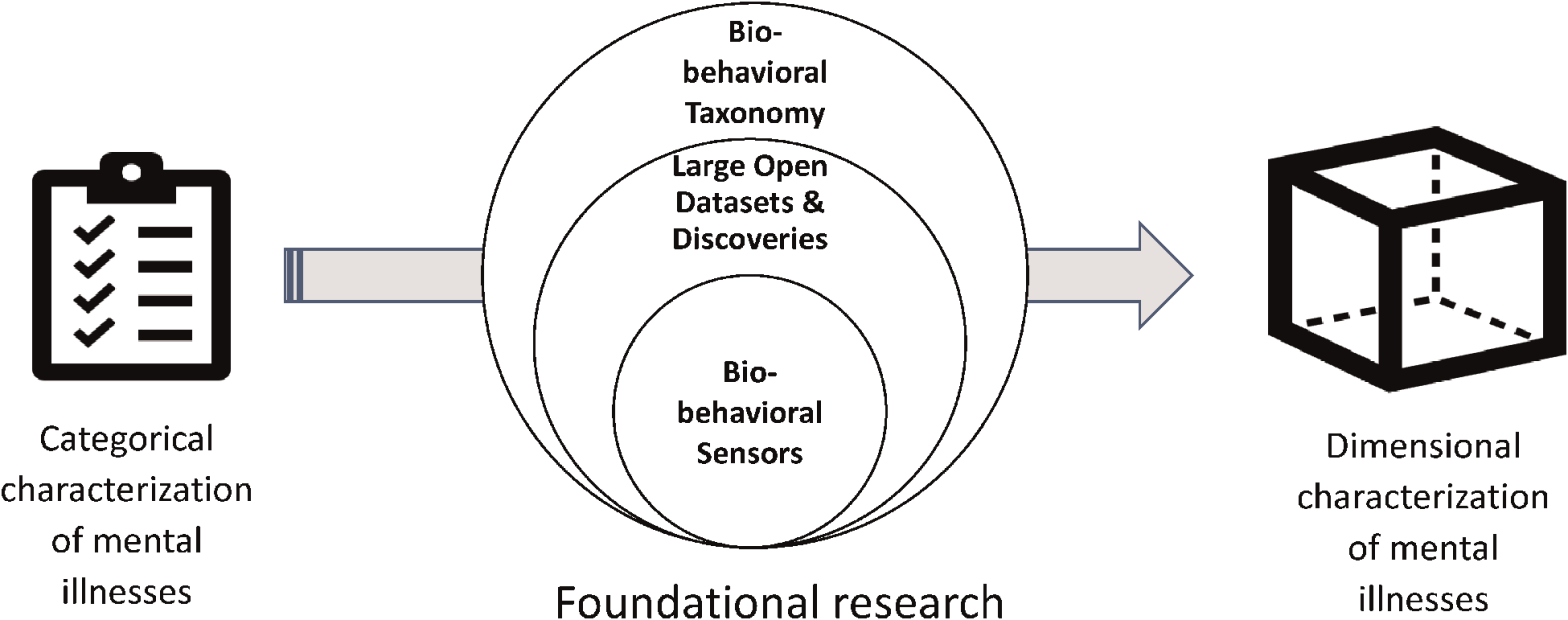
The role of big data in understanding mental health symptoms.

To effectively map sensor data to mental health constructs, there are some challenges to overcome (Figure 2). The main two are the absence of a pre-illness baseline, and the need to account for individual differences. Different patients manifest various signs of their symptoms and mood in different behaviors. Our previous studies have shown that one big machine learning model does not adapt to the heterogeneity of different users. The author's team has built personalized models to map sensor-based markers and some self-reported data into mood by considering individual similarities and differences in the relationships between contributing behaviors and physiology and mood and finetuned pre-trained machine learning models (38, 39), but this work needs to be reproduced on a larger scale that would allow treatment development and implementation in the future.

## Brain imaging for mental health and its relation to technology use

The use of several *in vivo* human brain imaging techniques (PET, MRI, etc.) has resulted in tremendous advances in our understanding of brain function and dysfunction. The examples are too many to list, but specifically in depression, we now understand the importance of the default mode network, specific sub-regions of the prefrontal cortex, and the anterior cingulate. This knowledge is slowly resulting in possible actionable results, such as optimizing antidepressant choice, and improving transcranial magnetic stimulation protocols. However, for several reasons, in the field of mental health, the application of these techniques to the clinic is still mainly in the promise stage.

In mental health, brain imaging parameters are likely to be used, in the future, as diagnostic and prognostic tools, and as tools to help personalize therapeutic approaches. To achieve that stage, it is necessary to find robust biomarkers that will allow to a) to differentiate between unipolar and bipolar mood disorder patients (thus helping with commonly confused diagnoses), b) to predict which patients are at higher risk of suicide upon leaving a clinic (thus helping with treatment intensity and resource allocation), or c) to help clinicians choose among different antidepressants for a specific patient instead of blindly trying drugs by trial and error for each patient.

The field of biomarkers in psychiatry is advancing on different sub-fields, such as genetics (40–42), epigenetics (43), and brain imaging (40, 44, 45). However, progress is very slow: Most reasons for death have decreased over time due to medical advances (cancer being an obvious example) while death by suicide has not decreased and in some cases has actually increased considerably (38, 46, 47). As an example, the likely two biggest pharmacological breakthroughs in mental health in the last ∼50 years have been the use of selective serotonin reuptake inhibitors (SSRIs, the first one was Prozac, which received FDA approval in 1987), and ketamine, almost 30 years later. Although it is unclear yet whether ketamine will become a widely used antidepressant, it rapid form of action has opened the possibility of stabilizing patients in a very short time, which may be important in some cases. Several groups are studying the possibility of chronic or semi-chronic use of ketamine, with promising results. Regardless of the future of ketamine use in the clinical setting, the fact remains that it was the major pharmacological novelty in the treatment of depression since the 80′s. The drawbacks of ketamine use (short half-life, short action duration, addiction potential) may make the molecule itself not sustainable as a first time long term treatment. However, the discovery and use of ketamine intravenously, intransally, and now in orla form in clinical trials, has spurred clinical trials of ketamine metabolites and compounds with similar GABA and glutamate-based action for depression. There are several possible reasons why this happens, and why we need to use wearable and smartphone data to advance biotyping and endophenotype refinement in psychiatry.

The most obvious reason why psychiatry advances slower than other medical fields is the fact that there are no biomarkers robust enough to be used clinically. This is probably because we don’t understand the functioning of the affected organ, so we have no way to test how this organ is working in a patient. Thus, psychiatry relies almost 100% on self-report which no other medical field does. Accordingly, many labs across the world are working on finding biomarkers using MRI and other techniques. A major issue that appears is the fact that psychiatric disorders are not true disorders but generally, just collections of symptoms that are at best loosely connected in a symptom cluster. For example, major depressive disorder (MDD) is defined in the Diagnostic and Statistical Manual of Mental Disorders, Fifth Edition (2) as having 5 out of 9 symptoms (with the necessary inclusion of one of the first two symptoms: depressed mood and/or anhedonia). This makes it possible that two patients with the same MDD diagnosis have no overlapping symptoms, and therefore different biotypes. Using functional imaging can be a way to tackle this diversity and improve both biotyping and treatment stratification. For example, biotyping for anhedonia allows linking to transcranial magnetic stimulation treatments (48). However as functional imaging is expensive, we argue that it can be used as ground truth, but needs to have data-based, sensor-linked correlates from the field in order to truly be scalable in use.

Another problem that is seldom mentioned is the lack of appropriate comparison control groups. For example, one of the most replicated biomarkers in psychiatry research is the fact that the volume of the hippocampus is smaller in MDD patients than in healthy controls (49, 50) including a study from the ENIGMA consortium including several thousands of participants (44). Although the volumetric difference is not enough to be used diagnostically, it is statistically extremely significant given a large number of participants and manuscripts that studied this question. However, the comparison between MDD patients and healthy controls is problematic: the comorbidity of psychological disorders is common, so it is difficult to describe the fact that these patients have a small hippocampus with depression, as it may be an effect of a number of possible (diagnosed or not) common comorbidities such as alcohol and drug use, high anxiety levels, etc. A common strategy for this problem is to recruit MDD patients (or patients of any other disorder) while excluding patients that have any other diagnosis and comparing those to healthy controls. This results in two problems: First, the samples are not tremendously representative, as comorbidities are common in psychiatry; and second, “hidden” variables may be driving the results, such as chronic stress, poor sleep patterns, or feelings of stigma. These are associated not only with MDD but with most other psychiatric disorders. To attack this problem, we studied hippocampal volume in a highly comorbid sample of psychiatric patients and compared MDD patients, healthy controls, and “psychiatric controls” (patients in the same clinic that are not MDD, but matched to the MDD group by sex, age, and all psychiatric comorbidities). The results showed that indeed MDD patients had smaller hippocampi than healthy controls, as published multiple times. However, we found no difference in hippocampal volume between MDD and psychiatric controls. Thus, MDD is not associated with smaller hippocampi, at least not in a specific manner. In addition, we showed that patients with other diagnoses, namely alcohol use disorder, borderline personality disorder, and post-traumatic stress disorder, showed smaller hippocampi when compared to healthy controls but not when compared with psychiatric controls (51–53). We postulate that the comparison between psychiatric patients and healthy controls is incorrect because it does not take into account variables either known or hypothesized to affect the measure of interest (such as hippocampal volume). Being that the scientific literature and many current projects still rely on comparing diagnosed patients to healthy controls, we believe that the chances of finding biomarkers that are specific for a certain group of patients are extremely small.

### The gap between functional imaging and technology-generated data is deep

For example, in relation to the clinical application mentioned below (cravings), findings from habenula research indicate that habenula connectivity relates to the extent of opioid use in psychiatric patients (54) through a sample of 305 patients (51 opioid users) from the Menninger Clinic psychiatric inpatient population. Using the World Health Organization ASSIST scale (assessing substance involvement and health risks), the team assessed patients as either opioid non-users or users (low vs. moderate/high-risk scores respectively). Given the habenular circuitry and the general model of addiction in which basal ganglia activity is one of the main drivers of the cycle of addiction (41), the team concentrated on the habenula, a small nucleus critical for the reward system, known to be involved in nicotine addiction. Since nicotinic and opioid receptors are highly co-expressed in the habenula, the team tested whether the habenula is a critical brain locus for opioid abuse. The primary outcome was resting-state functional connectivity (RSFC) between the habenula and the striatum. The team then subdivided the striatum into its parts: the caudate, putamen, and globus pallidus for further analysis and found increased right habenula/striatum RSFC in opioid users compared to non-users, especially in the caudate. The team showed that the connectivity between the caudate (more related to habits than to reward itself) and the habenula (control of negative prediction errors) is increased in opioid abusers. Thus, it could be theoretically possible to classify opioid users based on reward sensitivity. However, the link between this kind of data and behavioral data is not established, and is a missing link in the current research landscape.

The ability to discern the intensity of craving to establish “ground truth” for craving responses, using self-reports and associating fMRI activity that regresses onto self-reports, would be very helpful in this case and bypass difficulties in using fMRI on regular basis given the cost and other limitations.

There is, however, a reason for optimism and we believe psychiatric biomarker research will undoubtedly generate actionable results in the near future, when combined with properly analyzed sensor-based data. Research is not only advancing our understanding of brain mechanisms of function and misfunction, but also pointing to new and improved possible therapeutic approaches.

### Clinical application #1: Anhedonia

In this example, we will highlight how the spectrum of tools available from fMRI to wearable-generated data can be applied to clinical, dimensional hierarchical psychiatric concepts.

Anhedonia is the reduced ability to feel pleasure, and enjoy activities (55). It is a core symptom of depression but is also present in psychotic and addictive disorders. Anhedonia can be divided into anticipatory/motivational and consummatory (56), based on effects on reward processing.

Anticipatory anhedonia correlates with poor social outcomes in depression and can be responsive to connectivity-based transcranial magnetic stimulation treatments (48, 57), but may be less responsive to antidepressant treatments. Imaging studies define anhedonia as low reward processing, involving altered cortical thickness (58) with asymmetry in depressive and subthreshold depressive symptoms.

Anhedonia may be well defined in the mind of clinicians inquiring about it as a depressive symptom, but needs further definition and refined quantification as a dimension, and this is where multi-modal, interdisciplinary characterization can be particularly well-suited. The sociability techniques mentioned above, though still experimental, could serve as a bedrock for a conversation in the clinic discussing perceived social isolation versus actual social interactions as objectively measured.

Digital phenotyping of depression, using smartphone applications and wearables is firmly established as a developing, promising measure for depressive symptom (17, 42, 59–62), but has not specifically targeted anhedonia. In contrast to anhedonia, the field of negative symptoms in psychotic disorders is well developed. Mobility patterns *via* smartphones have been found to have discriminatory and quantification abilities in psychotic disorders, by detecting anhedonia and negative symptoms (59, 60). Avolition and anhedonia predict outcomes in social situations (56, 59, 60).

Throughout the day, an individual will have fluctuations in emotions, positive and negative, and make decisions about behaviors based on those emotions. The intensity of the emotion matters as well as the valence, thus a small fluctuation in a negative valence might be an adequate, environmentally-appropriate response to a stressful event (e.g. getting upset, distracted, maybe a little tearful in a discrete way after a criticism), but a negative reaction of large intensity may affect the individual's ability to function (sobbing uncontrollably in the bathroom). Negative affect might be depressive or anhedonic in nature*. All these events can be captured via phone/wearable sensing*. Digital markers of behavioral change (i.e. observable changes in sleep, physical activity, and social interaction) might become sensitive measures of meaningful variation in anhedonia, then related functional status. Did the patient interact with other people? Did they leave early? Did they end up not sleeping well that night, report auditory hallucinations, or other issues? All these EMA elements and sensor-based data can be collected in real-time, and now wait until a clinic visit, where recall could be subject to bias.

Another way digital data collection can serve with the clinical concept is anhedonia is a further refinement of what constitutes anhedonia to the assessing clinician versus the user/patient. Subjective anhedonia assessment may be culturally modulated (34, 63), and in an exploratory survey of individuals from various backgrounds, ecological momentary assessment (EMA) of depressed mood and anhedonia suggests that EMA of anhedonia and depressed mood are not always to be taken at face value, as patients may report feeling depressed as anhedonic, and have a poor distinction of low mood and the behavioral correlates attached to it, such as social avoidance, low engagement in activities, or mood swings. Higher levels of anhedonia relate to anxiety related to social situations (social anhedonia) (34), a preference to be alone or with people one is at ease with. Thus, measuring social interaction is essential to understanding anhedonia both on a personal and social levels, though the two constructs are not fully overlapping (dysfunctional reward processing (RDoC Positive Valence System versus RDoC Affiliation and Attachment subconstruct within Social Processes, for social anhedonia).

### Clinical application #2: Cravings

Craving is defined as the subjective experience of an urge or desire to use substances. The intensity of cravings predicts relapse even when individuals are engaged in inpatient treatment (64, 65). For opioids, cravings almost universally precede opioid use, and the intensity and timing of cravings are associated with the intensity of use (66, 67). When an individual is addicted to a substance (e.g. opioids, stimulants, alcohol), viewing a stimulus related to that substance increases cravings; Myrick showed, for instance, that “after a sip of alcohol and exposure to alcohol beverage pictures, alcoholics compared to social drinkers had increased differential brain activity in the prefrontal cortex and anterior thalamus” (68).

What is craving? Cravings result from the interplay of internal factors and external contextual/environmental factors. To define cravings, one must realize it is not a unified phenomenon but a complex construct, and a reflection of multiple brain processes (14). Advances in functional imaging have allowed a better definition of the craving phenomenon, but not a distinction of craving subtypes. Much is still not understood about how a craving develops, how it unfolds, and what factors modulate it. For the purposes of this discussion, we will divide factors into **internal** (mood, affect, feeling pain or withdrawal symptoms, being impulsive, physiological factors, etc.) and **contextual/environmental** factors (drug availability, support system, drug-using friends nearby, legal issues and other outside factors). Of note, some of these factors are short-term (where the person is at the moment of experiencing a craving) to long-term (having experienced trauma, having an injury, current stressors). The presence of co-occurring psychiatric disorders such as depression (69, 70), Post-traumatic stress disorder (PTSD), or anxiety may modulate cravings and associated affective intensity. In opioid users, negative affect induction worsens and promotes drug cravings (67, 71). Cravings are considered as short-lasting moments and are subjectively assessed (20), with no objective markers recognized as a gold standard for assessment and evaluation. However, it is unknown whether the impact of mood factors and physiological factors (such as stress and sleep issues) is cumulative, and if so, how. This could be a form of medium-term, yet undefined, sustained cravings state. Most of these changes can be collected and followed by a combination of EMA and sensor-based (wearable) detection. Other unknowns include how and when a craving leads to an actual relapse on the drug of choice for the user, with impulsivity and impaired temporal discounting being implicated as proxy measures for weakened self-regulation in the face of a craving. Other factors include pain, which could trigger cravings, and misuse or over-use of opioids (72, 73). Subjective feelings of opioid withdrawal, including anxiety, have been shown to predict cravings as well. GPS mobility patterns to predict opioid drug craving or stress 90 min into the future among patients with opioid use disorder with buprenorphine or methadone showed a positive predictive value of 0.93 (67). Thus, introducing wearable technology (or smartphone) could theoretically be used in real-time for cravings detection and mitigation.

Interestingly, cravings are often thought of as associated with stress and negative affect. Much like in the above example, anhedonia, using objective sensor-based data could help refine the concept of cravings. While negative affect is certainly pertinent to a majority of relapse situations given increased stress vulnerability in addictive disorders in general, stress is not a necessary requirement for cravings. Some cravings relapses are thought to occur in the spirit of reward-seeking (66, 74, 75). In individuals with OUD, for instance, prevalence of non-substance addictive behaviors such as binge eating, hypersexual behavior, excessive video gaming, and gambling is up to 47% and is heavily linked to impulsivity (76, 77). Wearable technology could also help detect these behaviors and paint a more complete picture of cravings and their behavioral correlates. Of note, functional imaging has been heavily used in craving studies, but not used to biotype cravings or guide treatment, an open opportunity in this field.

**Figure 3:**
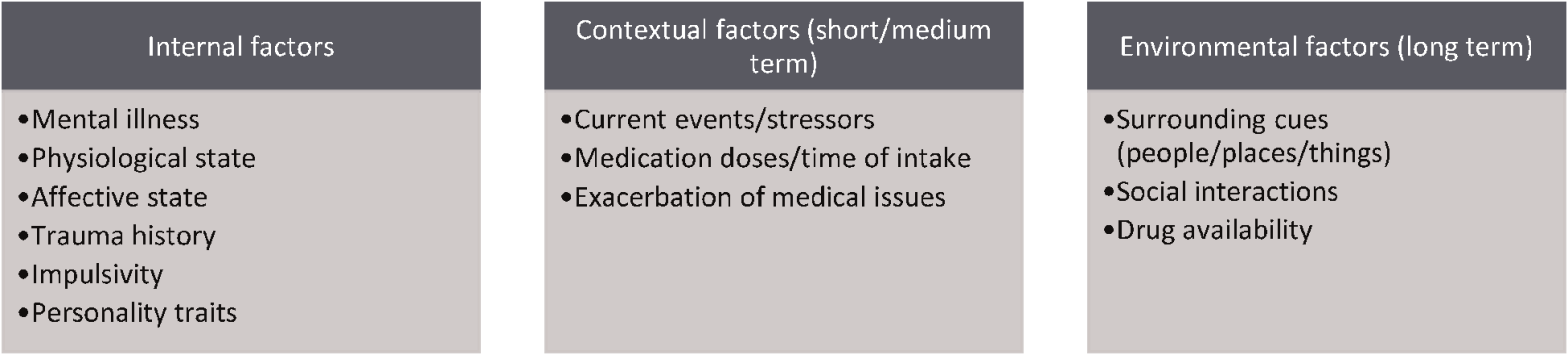
Factors potentially contributing to cravings and measurable by wearable devices.

The interaction between external factors (commonly summed in the literature as external cues, with the process of cue-induced reactivity as a trigger to relapse), and internal factors of short, medium, and long-term nature may or may not result in the use of drugs (relapse). As individuals become more advanced in drug abstinence/sobriety, they acquire a better ability to manage cravings and affective correlates of cravings such as stress while simultaneously learning to redirect their energy to non-drug use behaviors. Figure 3 summarizes the spectrum of factors thought to be related to cravings. A properly designed technology framework ought to capture the progression of emotional states signaling potential relapse, detect behavioral changes indicating deviation from the patient's baseline, and ideally, suggest an ecological momentary intervention to prevent a relapse.

## Balancing innovation with patient privacy and comfort

The discussion in previous sections highlights the diversity of ongoing research to both better understand mental health conditions quantitatively and use that information to develop novel targeted interventions. Two large classes of data are being considered. First is biological data, e.g., brain imaging discussed in the previous section. Since most healthcare relies on biological data, ranging from stethoscope use in a doctor's office to diverse imaging and blood analyses, most patients are comfortable with the idea of sharing such information with their healthcare providers. Clinical workflows are well established for sharing, storing, analyzing, and reporting biological data.

However, the second form of data that relates to patient emotions and behaviors (activity, sleep, sociability, work-related, location, etc.), as discussed in Section II, has little or no precedence in current healthcare systems. At a personal level, we (at least partially) define our identity by our behaviors—“I am me because of what I do throughout the day.” Perhaps even more important is that we share a version of ourselves with others, which is often better than reality and indicative of how we want others to view us—we want others to view us positively. By allowing sensors to be attached to us, tracking our reality without “sugar coating” it, and thereby exposing what we really do, can be highly unsettling for many. Coupled with this potential patient barrier is the fact that these forms of data are so new that we have little well-formed evidence on how to interpret behavioral data for each patient and use them in clinical interventions. Additionally, there will be a significant practical challenge in training clinicians to avoid judgement on patient discordance, when patient sensor data tells a story that is divergent from patient self-view; e.g., “I exercise regularly” but the data shows otherwise.

We believe that the solution lies in four layers. First, we should recognize that potential discordance between measurable reality and its representation in our brains is natural. We should recognize that our brains are not designed for accurately counting large numbers or accurately sensing time. Thus, if we cannot estimate how much we exercised, then it is not because we plan to lie about it but because we are simply not good at it. By recognizing that all humans face this limitation ensures that no one feels deficient as part of their health condition. Recognizing this fundamental human limitation will both lower the barrier in patient acceptance and at the same time, eliminate any clinical bias. At the same time, clinicians will find that there is clinical value in identifying the level of discordance in some conditions. For example, for patients with psychotic disorders, the level of discordance may be an indicator of medication effectiveness.

Second, it is important that we standardize how behavioral data will be processed and we share that information with patients, so that the patients can form an informed judgement. For example, “the daily step count will help us study trends in physical activity, since improved physical activity is associated with improved response to depression.”

Third, we should investigate analysis methods that can extract quality-of-life parameters from quantity measures, which could further improve patient confidence that no deeply private information is being analyzed. For example, our recent work on using free-form speech recording to evaluate social ambiance performs no speech content analysis. Instead, it uses the number of concurrent speakers to estimate how socially busy or isolated the patient environment is throughout the day. The methods thus aim to estimate sociability from the number of simultaneous speakers and completely side-steps speech content analysis; as a by-product, it is language independent and can be applied to multi-lingual environments.

Finally, new engineering innovations are increasingly able to support the above desirable properties of data analysis and protected storage. For example, wearables like smartwatches now have powerful computing resources to process raw data (e.g., bio-markers or speech) on the device itself and extract only the data features that are needed for clinical analysis. Combined with end-to-end encryption, these extracted features can be securely transported and stored, in ways such that the service provider (e.g., cloud computing provider) cannot view any of the data.

An overall societal trend is also shaping how future generations may perceive these advanced techniques. Each generation seems to be openly sharing more about themselves, and thereby changing societal norms of what is acceptable to share. While we lack an individual baseline at this point, the time may come when every person has a recorded healthy “digital baseline” that can be used if they fall ill.

This expanding boundary is playing positively towards supporting the ambitious research agenda discussed in this paper. However, it does not forego our responsibility to protect patient data and privacy, as patient confidence is fundamental in acceptance of medical treatment and overall adherence.

## Author contributions

NM – design and writing of the manuscript (primary), AS – design and writing of the manuscript + (manuscript + figures), RS – design and writing of the manuscript (section 2/3), ZH – design and writing of the manuscript (sections 3/4), AS – design and writing of the manuscript + overall editing and conceptualization. All authors contributed to the article and approved the submitted version.

## References

[1] Couper MP Gremel G Axinn W Guyer H Wagner J West BT. New options for national population surveys: The implications of internet and smartphone coverage. Soc Sci Res. (2018) 73:221–35. 10.1016/j.ssresearch.2018.03.008 29793688 PMC10799630

[2] Association AP. Diagnostic and statistical manual of mental disorders (DSM-5®). 5th ed. Washington: American Psychiatric Pub (2013). 10.1176/appi.books.9780890425596

[3] WHO. International statistical classification of diseases and related health problems. 11th ed. Geneva: Switzerland (2019). https://icd.who.int/browse11

[4] Arseneault L. Taxonomy of psychopathology: A work in progress and a call for interdisciplinary research. World Psychiatry. (2021) 20(1):73–4. 10.1002/wps.20817 33432752 PMC7801852

[5] Low DM Bentley KH Ghosh SS. Automated assessment of psychiatric disorders using speech: A systematic review. Laryngoscope Investig Otolaryngol. (2020) 5(1):96–116. 10.1002/lio2.354 32128436 PMC7042657

[6] Low DM Rumker L Talkar T Torous J Cecchi G Ghosh SS. Natural language processing reveals vulnerable mental health support groups and heightened health anxiety on reddit during COVID-19: Observational study. J Med Internet Res. (2020) 22(10):e22635. 10.2196/22635 32936777 PMC7575341

[7] Dibeklioglu H Hammal Z Cohn JF. Dynamic multimodal measurement of depression severity using deep autoencoding. IEEE J Biomed Health Inform. (2018) 22(2):525–36. 10.1109/JBHI.2017.2676878 28278485 PMC5581737

[8] Kacem A Hammal Z Daoudi M Cohn JF. Detecting depression severity by interpretable representations of motion dynamics. 13th IEEE International Conference on Automatic Face & Gesture Recognition, FG 2018; May 15–19, 2018; Xi’an, China (2018). p. 739–745 10.1109/FG.2018.00116 PMC6157749 30271308

[9] Scherer S Hammal Z Yang Y Morency L-P Cohn JF. Dyadic behavior analysis in depression severity assessment interviews. Proceedings of the 16th International Conference on Multimodal Interaction, ser. ICMI ‘14; New York, NY, USA: ACM (2014). p. 112–119. Available at http://doi.acm.org/10.1145/2663204.2663238 10.1145/2663204.2663238 PMC5365085 28345076

[10] Alghowinem S Goecke R Wagner M Parkerx G Breakspear M. Head pose and movement analysis as an indicator of depression. Affective computing and d Intelligent Interaction (ACII), 2013 Humaine association Conference on (2013). p. 283–288

[11] Joshi J Goecke R Parker G Breakspear M. Can body expressions contribute to automatic depression analysis? 2013 10th IEEE International Conference and Workshops on Automatic Face and Gesture Recognition (FG); April 2013 (2013). p. 1–7

[12] Daoudi M Hammal Z Kacem A Cohn JF. Gram matrices formulation of body shape motion: an application for depression severity assessment. 2019 8th International Conference on Affective Computing and Intelligent Interaction Workshops and Demos (ACIIW); 2019, September: IEEE (2019). p. 258–63

[13] Pampouchidou A, Olympia S, Amir F, Matthew P, Dimitris M, Alexandros R, Georgios G, et al. Automatic assessment of depression based on visual cues: A systematic review. EEE Trans Affect Comput. (2017) 10(4):445–70. 10.1109/TAFFC.2017.2724035

[14] https://www.frontiersin.org/articles/10.3389/fpsyt.2021.670020/full.

[15] Lamichhane B, Moukaddam N, Patel AB, Sabharwal A. ECoNet: Estimating Everyday Conversational Network From Free-Living Audio for Mental Health Applications. *IEEE Pervasive Computing*. (2022).

[16] Seppälä J De Vita I Jämsä T Miettunen J Isohanni M Rubinstein K Mobile phone and wearable sensor-based mHealth approaches for psychiatric disorders and symptoms: Systematic review. JMIR Ment Health. (2019) 6(2):e9819. 10.2196/mental.9819 30785404 PMC6401668

[17] Moukaddam N Truong A Cao J Shah A Sabharwal A. Findings from a trial of the smartphone and OnLine usage-based eValuation for depression (SOLVD) application: What do apps really tell US about patients with depression? Concordance between app-generated data and standard psychiatric questionnaires for depression and anxiety. J Psychiatr Pract. (2019) 25(5):365–73. 10.1097/PRA.0000000000000420 31505521

[18] Cao J Truong AL Banu S Shah AA Sabharwal A Moukaddam N. Tracking and predicting depressive symptoms of adolescents using smartphone-based self-reports, parental evaluations, and passive phone sensor data: Development and usability study. JMIR Ment Health. (2020) 7(1):e14045. 10.2196/14045 32012072 PMC7007590

[19] Crawford JR Henry JD. The positive and negative affect schedule (PANAS): Construct validity, measurement properties and normative data in a large non-clinical sample. Br J Clin Psychol. (2004) 43(3):245–65. 10.1348/0144665031752934 15333231

[20] Crawford K Paglen T. Excavating AI: The politics of images in machine learning training sets. AI Soc. (2021):1–12. 10.1007/s00146-021-01162-8

[21] John OP Naumann LP Soto CJ. Paradigm shift to the integrative big five trait taxonomy: History, measurement, and conceptual issues. Washington (2008).

[22] Cohen S Janicki-Deverts D. Who's stressed? Distributions of psychological stress in the United States in probability samples from 1983, 2006, and 2009 1. J Appl Soc Psychol. (2012) 42(6):1320–34. 10.1111/j.1559-1816.2012.00900.x

[23] Ware JE Jr Kosinski M Keller SD. A 12-item short-form health survey: Construction of scales and preliminary tests of reliability and validity. Med Care. (1996) 34(3):220–33. 10.1097/00005650-199603000-00003 8628042

[24] Taylor S Jaques N Chen W Fedor S Sano A Picard R. Automatic identification of artifacts in electrodermal activity data. Paper presented at: 2015 37th Annual International Conference of the IEEE Engineering in Medicine and Biology Society (EMBC) (2015). 10.1109/EMBC.2015.7318762. PMC5413200 26736662

[25] Sano A Taylor S McHill AW Phillips AJ Barger LK Klerman E Identifying objective physiological markers and modifiable behaviors for self-reported stress and mental health status using wearable sensors and mobile phones: Observational study. J Med Internet Res. (2018) 20(6):e210. doi: 10.2196/jmir.9410 29884610 PMC6015266

[26] Boonstra TW Nicholas J Wong QJ Shaw F Townsend S Christensen H. Using mobile phone sensor technology for mental health research: Integrated analysis to identify hidden challenges and potential solutions. J Med Internet Res. (2018) 20(7):e10131. 10.2196/10131 30061092 PMC6090171

[27] Jaques N Taylor S Azaria A Ghandeharioun A Sano A Picard R. Predicting students’ happiness from physiology, phone, mobility, and behavioral data. Paper presented at: 2015 International Conference on Affective Computing and Intelligent Interaction (ACII) (2015). 10.1109/ACII.2015.7344575. PMC5431070 28515966

[28] Tseng VW Sano A Ben-Zeev D Brian R Campbell AT Hauser M Using behavioral rhythms and multi-task learning to predict fine-grained symptoms of schizophrenia. Sci Rep. (2020) 10(1):15100. 10.1038/s41598-020-71689-1 32934246 PMC7492221

[29] Zhou J, Lamichhane B, Ben-Zeev D, Campbell A, Sano A. Predicting psychotic relapse in schizophrenia with mobile sensor data: routine cluster analysis. *JMIR Mhealth Uhealth*. (2022) 10(4):e31006. 10.2196/31006 PMC9039818 35404256

[30] Lamichhane B, Zhou J, Sano A. Psychotic Relapse prediction in schizophrenia patients using a mobile sensing-based supervised deep learning model. arXiv preprint arXiv:2205.12225. 10.1109/JBHI.2023.3265684 37037254

[31] Elmer T Mepham K Stadtfeld C. Students under lockdown: Comparisons of students’ social networks and mental health before and during the COVID-19 crisis in Switzerland. PloS One. (2020) 15(7):e0236337. 10.1371/journal.pone.0236337 32702065 PMC7377438

[32] Pinquart M Sörensen S. Influences on loneliness in older adults: A meta-analysis. Basic Appl Soc Psych. (2001) 23:245–66. 10.1207/S15324834BASP2304_2

[33] Boldrini T Tanzilli A Di Cicilia G, Gualco I, Lingiardi V, Salcuni S, et al. Personality traits and disorders in adolescents at clinical high risk for psychosis: Toward a clinically meaningful diagnosis. Front Psychiatry. (2020) 11:562835. 10.3389/fpsyt.2020.562835 33363479 PMC7753018

[34] Brown LH Silvia PJ Myin-Germeys I Kwapil TR. When the need to belong goes wrong: The expression of social anhedonia and social anxiety in daily life. Psychol Sci. (2007) 18(9):778–82. 10.1111/j.1467-9280.2007.01978.x 17760772

[35] Gianfredi V Beran M Koster A, Eussen SJ, Odone A, Signorelli C, et al. Association between social network characteristics and prevalent and incident depression: The maastricht study. J Affect Disord. (2021) 293:338–46. 10.1016/j.jad.2021.06.046 34229287

[36] Chen W. AmbianceCount: an objective social ambiance measure from unconstrained day-long audio recordings. Houston, Texas: Rice University (2020). https://hdl.handle.net/1911/109640

[37] Falkenström F Solomonov N Rubel J. Using time-lagged panel data analysis to study mechanisms of change in psychotherapy research: Methodological recommendations. Couns Psychother Res. (2020) 20(3):435–41. 10.1002/capr.12293 34093088 PMC8171261

[38] Blanco C Hoertel N Franco S, Olfson M., He JP, Lopez S, et al. Generalizability of clinical trial results for adolescent Major depressive disorder. Pediatrics. (2017) 140(6):e20161701. 10.1542/peds.2016-1701 29097612 PMC5703774

[39] Li B Sano A. Early versus late modality fusion of deep wearable sensor features for personalized prediction of tomorrow's mood, health, and stress. Annual International Conference of the IEEE Engineering in Medicine and Biology Society IEEE Engineering in Medicine and Biology Society Annual International Conference; 2020 (2020). p. 5896–9. 10.1109/EMBC44109.2020.9175463 33019316

[40] Bielczyk NZ Buitelaar JK Glennon JC Tiesinga PH. Circuit to construct mapping: A mathematical tool for assisting the diagnosis and treatment in major depressive disorder. Front Psychiatry. (2015) 6:29. 10.3389/fpsyt.2015.00029 25767450 PMC4341511

[41] Leuchter AF Hunter AM Krantz DE Cook IA. Intermediate phenotypes and biomarkers of treatment outcome in major depressive disorder. Dialogues Clin Neurosci. (2014) 16(4):525–37. 10.31887/DCNS.2014.16.4/aleuchter 25733956 PMC4336921

[42] Melcher J Hays R Torous J. Digital phenotyping for mental health of college students: A clinical review. Evid Based Ment Health. (2020) 23(4):161–6. 10.1136/ebmental-2020-300180 32998937 PMC10231503

[43] Pitsillou E Liang J Hung A Karagiannis TC. The circadian machinery links metabolic disorders and depression: A review of pathways, proteins and potential pharmacological interventions. Life Sci. (2021) 265:118809. 10.1016/j.lfs.2020.118809 33249097

[44] Frangou S Modabbernia A Williams SCR Papachristou E Doucet GE Agartz I Cortical thickness across the lifespan: Data from 17,075 healthy individuals aged 3–90 years. Hum Brain Mapp. (2021) 389–408. 10.1002/hbm.25364 PMC8675431 33595143

[45] Zhang D Gao J Yan X Tang M Zhe X Cheng M Altered functional connectivity of brain regions based on a meta-analysis in patients with T2DM: A resting-state fMRI study. Brain Behav. (2020) 10(8):e01725. 10.1002/brb3.1725 32558376 PMC7428490

[46] Hyman S Chisholm D Kessler R Patel V Whiteford H. Mental disorders. In: Jamison DT Breman JG, editors. Disease control priorities in developing countries. Washington, DC: World Bank The International Bank for Reconstruction and Development/The World Bank Group (2006). p. 1–6. Bookshelf ID: NBK11728. 21250309

[47] Williams SB O'Connor EA Eder M Whitlock EP. Screening for child and adolescent depression in primary care settings: A systematic evidence review for the US preventive services task force. Pediatrics. (2009) 123(4):e716–735. 10.1542/peds.2008-2415 19336361

[48] Wang X He K Chen T Shi B Yang J Geng W Therapeutic efficacy of connectivity-directed transcranial magnetic stimulation on anticipatory anhedonia. Depress Anxiety. (2021) 38(9):972–84. 10.1002/da.23188 34157193

[49] Hastings RS Parsey RV Oquendo MA Arango V Mann JJ. Volumetric analysis of the prefrontal cortex, amygdala, and hippocampus in major depression. Neuropsychopharmacology. (2004) 29(5):952–9. 10.1038/sj.npp.1300371 14997169

[50] Masi G Brovedani P. The hippocampus, neurotrophic factors and depression. CNS Drugs. (2011) 25(11):913–31. 10.2165/11595900-000000000-00000 22054117

[51] Gosnell S Fowler J Salas R. Classifying suicidal behavior with resting-state functional connectivity and structural neuroimaging. Acta Psychiatr Scand. (2019) 140(1):20–9. 10.1111/acps.13029 30929253

[52] Poblete GF Gosnell SN Meyer M Fang M Nguyen T Patriquin MA Process genes list: An approach to link genetics and human brain imaging. J Neurosci Methods. (2020) 339:108695. 10.1016/j.jneumeth.2020.108695 32201351

[53] Avelar-Pereira B Bäckman L Wåhlin A Nyberg L Salami A. Increased functional homotopy of the prefrontal cortex is associated with corpus callosum degeneration and working memory decline. Neurobiol Aging. (2020) 96:68–78. 10.1016/j.neurobiolaging.2020.08.008 32949903

[54] Salas R Baldwin P de Biasi M Montague PR. BOLD Responses to negative reward prediction errors in human habenula. Front Hum Neurosci. (2010) 4:36. 10.3389/fnhum.2010.00036 20485575 PMC2872503

[55] Ryan ND Puig-Antich J Ambrosini P Rabinovich H Robinson D Nelson B The clinical picture of major depression in children and adolescents. Arch Gen Psychiatry. (1987) 44(10):854–61. 10.1001/archpsyc.1987.01800220016003 3662742

[56] Reilly EE Whitton AE Pizzagalli DA Rutherford AV Stein MB Paulus MP Diagnostic and dimensional evaluation of implicit reward learning in social anxiety disorder and major depression. Depress Anxiety. (2020) 37(12):1221–30. 10.1002/da.23081 32906219

[57] Tadros A Layman SM Davis SM Davidov DM Cimino S. Emergency visits for prescription opioid poisonings. J Emerg Med. (2015) 49(6):871–7. 10.1016/j.jemermed.2015.06.035 26409674 PMC4760637

[58] Dotson VM Taiwo Z Minto LR Bogoian HR Gradone AM. Orbitofrontal and cingulate thickness asymmetry associated with depressive symptom dimensions. Cogn Affect Behav Neurosci. (2021) 24:1297–1305. 10.3758/s13415-021-00923-8 34136976

[59] Harvey PD Khan A Keefe RSE. Using the positive and negative syndrome scale (PANSS) to define different domains of negative symptoms: Prediction of everyday functioning by impairments in emotional expression and emotional experience. Innov Clin Neurosci. (2017) 14(11–12):18–22. PMID: , PMCID: 29410933 PMC5788247

[60] Raugh IM James SH Gonzalez CM Chapman HC Cohen AS Kirkpatrick B Geolocation as a digital phenotyping measure of negative symptoms and functional outcome. Schizophr Bull. (2020) 46(6):1596–607. 10.1093/schbul/sbaa121 32851401 PMC7751192

[61] Sequeira L Perrotta S LaGrassa J Merikangas K Kreindler D Kundur D Mobile and wearable technology for monitoring depressive symptoms in children and adolescents: A scoping review. J Affect Disord. (2020) 265:314–24. 10.1016/j.jad.2019.11.156 32090755

[62] Torous J Keshavan M. A new window into psychosis: The rise digital phenotyping, smartphone assessment, and mobile monitoring. Schizophr Res. (2018) 197:67–8. 10.1016/j.schres.2018.01.005 29338959

[63] Chentsova-Dutton YE Choi E Ryder AG Reyes J. “I felt sad and did not enjoy life”: Cultural context and the associations between anhedonia, depressed mood, and momentary emotions. Transcult Psychiatry. (2015) 52(5):616–35. 10.1177/1363461514565850 25603917

[64] Anton RF. What is craving? Models and implications for treatment. Alcohol Res Health. (1999) 23(3):165–73. PMCID: , PMID: 10890811 PMC6760371

[65] Bottlender M Soyka M. Impact of craving on alcohol relapse during, and 12 months following, outpatient treatment. Alcohol Alcohol. (2004) 39(4):357–61. 10.1093/alcalc/agh073 15208171

[66] Antons S Brand M Potenza MN. Neurobiology of cue-reactivity, craving, and inhibitory control in non-substance addictive behaviors. J Neurol Sci. (2020) 415:116952. 10.1016/j.jns.2020.116952 32534370

[67] Epstein DH Tyburski M Kowalczyk WJ Burgess-Hull AJ Phillips KA Curtis BL Prediction of stress and drug craving ninety minutes in the future with passively collected GPS data. NPJ Digit Med. (2020) 3:26. 10.1038/s41746-020-0234-6 32195362 PMC7055250

[68] Myrick H Anton RF Li X Henderson S Drobes D Voronin K Differential brain activity in alcoholics and social drinkers to alcohol cues: Relationship to craving. Neuropsychopharmacology. (2004) 29(2):393–402. 10.1038/sj.npp.1300295 14679386

[69] Elman I Borsook D. The failing cascade: Comorbid post traumatic stress- and opioid use disorders. Neurosci Biobehav Rev. (2019) 103:374–83. 10.1016/j.neubiorev.2019.04.023 31063739

[70] Fathi HR Yoonessi A Khatibi A Rezaeitalab F Rezaei-Ardani A. Crosstalk between sleep disturbance and opioid use disorder: A narrative review. Addict Health. (2020) 12(2):140–58. 10.22122/ahj.v12i2.249 32782736 PMC7395935

[71] Willner-Reid J Whitaker D Epstein DH Phillips KA Pulaski AR Preston KL Cognitive-behavioural therapy for heroin and cocaine use: Ecological momentary assessment of homework simplification and compliance. Psychol Psychother. 2016;89(3):276–93. 10.1111/papt.12080 26530031 PMC6193475

[72] MacLean RR Spinola S Manhapra A Sofuoglu M. Systematic review of pain severity and opioid craving in chronic pain and opioid use disorder. Pain Med. (2020) 21(2):e146–63. 10.1093/pm/pnz228 32034413

[73] Pergolizzi JV Jr Raffa RB Rosenblatt MH. Opioid withdrawal symptoms, a consequence of chronic opioid use and opioid use disorder: Current understanding and approaches to management. J Clin Pharm Ther. (2020) 45(5):892–903. 10.1111/jcpt.13114 31986228

[74] Alonso-Caraballo Y Guha SK Chartoff EH. The neurobiology of abstinence-induced reward-seeking in males and females. Pharmacol Biochem Behav. (2021) 200:173088. 10.1016/j.pbb.2020.173088 33333134 PMC7796946

[75] Dennis LE Kohno M McCready HD Schwartz DL Schwartz B Lahna D Neural correlates of reward magnitude and delay during a probabilistic delay discounting task in alcohol use disorder. Psychopharmacology. (2020) 237(1):263–78. 10.1007/s00213-019-05364-3 31673722 PMC6991625

[76] Heinz A Kiefer F Smolka MN Endrass T Beste C Beck A Addiction research consortium: Losing and regaining control over drug intake (ReCoDe)-from trajectories to mechanisms and interventions. Addict Biol. (2019):e12866. 10.1111/adb.12866 31859437

[77] Kriegler J Wegener S Richter F Scherbaum N Brand M Wegmann E. Decision making of individuals with heroin addiction receiving opioid maintenance treatment compared to early abstinent users. Drug Alcohol Depend. (2019) 205:107593. 10.1016/j.drugalcdep.2019.107593 31634665

